# The complete chloroplast genome of the genus *Azadirachta*

**DOI:** 10.1080/23802359.2022.2095230

**Published:** 2022-07-14

**Authors:** Si-Teng He, Jiang-Chong Wu, Yi-Xing Zheng, Yan-Ping Zhang, Shu-Hui Li, Xing-Min Peng

**Affiliations:** a Institute of Highland Forest Science, Chinese Academy of Forestry (CAF), Kunming, China; b Nanjing Forestry University, Nanjing, China; c Pu’er Forest Ecosystem Research Station, National Forestry and Grassland Administration of China, Pu’er, China; d Forestry and Grassland Research Institute of Liangshan Yi Autonomous Prefecture, Xichang, China

**Keywords:** Chloroplast genome, *Azadirachta indica*, *Azadirachta indica* var. *siamensis*, *Azadirachta excelsa*, Meliaceae

## Abstract

*Azadirachta* consists of 2 species and 1 variety indigenous to the tropical areas of the Indo-Malayan region. They are evergreen trees for multi-purpose utilization featured by containing azadirachtin. The complete chloroplast (cp) genome of *Azadirachta indica*, *A. indica* var. *siamensis* and *Azadirachta excelsa* were reported in this study, which was 160,876 bp, 160,477 bp, and 160,361 bp in length respectively. The whole cp genomes encode 131 genes (37 tRNA genes, 8 rRNA genes, and 86 protein-coding genes) in both *A. indica* and *A. excelsa*, while *A. indica* var. *siamensis* do not have the *rrn4.5S* gene in the inverted repeat regions. The phylogenetic analysis indicated that *A. indica* var. *siamensis* and *A. exselsa* were closely related and *A. indica* was separated from these two species, which suggested that *A. siamensis* could be a species rather than a variety.

## Introduction

The genus *Azadirachta* (Meliaceae) was established by the French botanist Adrien Henri de Jussieu in 1830, including 2 species and 1 variety which are usually called neem indigenous to the Indo-Malayan region (Peng et al. 2012). They are tropical evergreen trees that grow up to a height of 15–50 m. Their names are *Azadirachta indica* A. Juss., *A. indica* A. Juss. var. *siamensis* Valeton, and *Azadirachta excelsa* (Jack) M. Jacobs (Multilingual Multiscript Plant Name Database, http://www.plantnames.unimelb.edu.au). *A. indica*, usually called Indian neem, is originated from Myanmar and southern India; *A. indica* var. *siamensis*, usually called Thai neem indigenous to Thailand, also exists in neighboring countries, such as Myanmar, southern Laos and western Cambodia; while *A. excelsa*, usually called Philippine neem, distributes in southern Thailand, Philippines, and Malaysia (Schmutterer 1995). Their seed kernels are rich in the phytochemical known as azadirachtin (CAS:11141–17–6), which is a kind of highly active insect antifeedant against more than 400 pest species (Jarvis et al. 1999). In addition to being raw materials for environmental-friendly biological pesticides, plants of *Azadirachta* are used for dental care (Agrawal et al. 2020), arid-hot valley greening (Zheng et al. 2018), and etc. *A. indica* is the most widely used species of the genus, it has been introduced in more than 70 countries and regions, mainly in the arid tropical and subtropical areas of Asia, Africa, the Americas, Australia and the South Pacific islands, and approximately 91 million individuals have been planted (Schmutterer 1995; Koul 2004). An attempt by our group was made to cross *A. indica* and *A. indica* var. *siamensis* to breed new varieties, but artificial reciprocal pollination was hard to produce mature fruit (unpublished data), probably indicating some degree of reproductive isolation. The complete chloroplast (cp) genome of *A. indica* var. *siamensis* and *A. excelsa* have not been reported to date. In this study, we report the complete chloroplast genome of *Azadirachta*, including *A. indica, A. indica* var. *siamensis* and *A. excelsa*. Based on the full chloroplast information of this genus, it is helpful to better understand the interspecific relationships.

## Materials and methods

The fresh leaves of *A. indica, A. indica* var. *siamensis*, and *A. excelsa* were collected from a germplasm garden of the Institute of Highland Forest Science, Chinese Academy of Forestry in Yuanjiang County, Yunnan Province, southwest China (23°36′11″ N, 102°00′46″ E, elevation 420 m), and dried using silica gel. The collection of plant material was carried out in accordance with guidelines provided by the Institute of Highland Forest Science, CAF, and was also complied with the permission from the local Forestry Bureau. The specimens were identified by Si-Teng He (sitenghe@caf.ac.cn) and then deposited at the herbarium of the Institute of Highland Forest Science, CAF under the voucher number 20210420YL, 20210420TL, and 20210420FL. Genomic DNA was extracted from ∼4 mg dried leaf using Plant Genomic DNA Kit (Tiangen Biotech, Beijing, China). DNA sequencing was performed on the Illumina Novaseq platform (Illumina, San Diego, USA). The raw data were generated and used for *de novo* cp genome assembly with GetOrganelle (Jin et al. 2020). The predicted genes were annotated with CPGAVAS2 (Shi et al. 2019). The newly annotated complete cp genomes were submitted to GenBank with accession numbers OK037100, OK037101, and OK037102 assigned to *A. excelsa*, *A. indica* var. *siamensis*, and *A. indica*, respectively.

**Figure 1.**
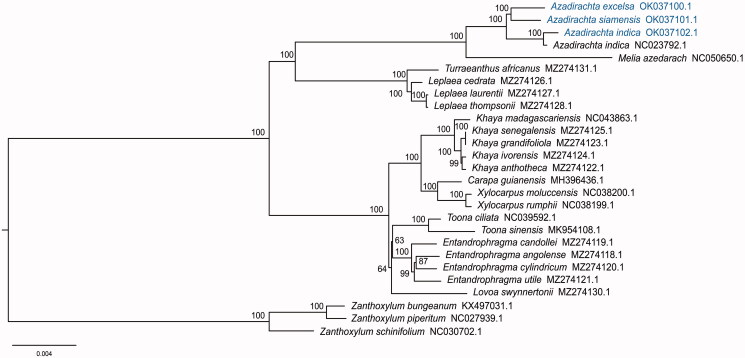
The Maximum-Likelihood (ML) phylogram of *Azadirachta* is inferred from the chloroplast genome sequences. Numbers at nodes correspond to the ML bootstrap support rate (1,000 replicates).

To reveal the phylogenetic position of *A. indica, A. indica* var. *siamensis*, and *A. excelsa*, the cp genomes of previously reported *A. indica* (GenBank NC023792.1) and 23 species from other genera of Meliaceae were downloaded and their GenBank accession numbers are provided in Figure 1. Three species from Rutaceae (*Zanthoxylum schinifolium*, *Z. bungeanum*, and *Z. piperitum*) were used as the outgroups. Preliminary multiple sequence alignment was performed using the online version MAFFT v7.490 (Katoh et al. 2019). Phylogenomic analysis was performed with the maximum likelihood (ML) method using RAxML v8.2.10 (Stamatakis 2014).

## Results

The complete cp genomes of these three *Azadirachta* plants are small circular DNA molecules with the typical quadripartite structure of land plant cp genomes consisting of a pair of inverted repeats (IRs) separated by large (LSC) and small (SSC) single copy regions. The GC content of the cp genomes of *A. indica*, *A. indica* var. *siamensis*, and *A. excelsa* are 37.496%, 37.540%, and 37.543%, respectively. These cp genomes are 160,876 bp in length encoding 131 genes (37 tRNA genes, 8 rRNA genes, and 86 protein-coding genes) in *A. indica*, 160,477 bp in length encoding 129 genes (37 tRNA genes, 6 rRNA genes, and 86 protein-coding genes) in *A. indica* var. *siamensis*, and 160,361 bp in length encoding 131 genes (37 tRNA genes, 8 rRNA genes, and 86 protein-coding genes) in *A. excelsa*, respectively. The length of LSC, SSC, and IR are 72,739 bp, 18,627 bp, and 27,054 bp respectively in *A. indica*; 72,689 bp, 18,649 bp, and 27,018 bp respectively in *A. indica* var. *siamensis*; and 72,696 bp, 18,594 bp, and 27,049 bp respectively in *A. excelsa*. Based on gene functions, the genes could be divided into four categories, self-replication genes, genes for photosynthesis, genes related to the biosynthesis of amino acids, fatty acids, and carboxylates, and functionally unknown genes (*ycf1*, *ycf2*, and *ycf4*). Among these genes, 18 genes are duplicated within the IRs of both *A. indica* and *A. excelsa*, including seven protein-coding genes (*rpl2*, *rpl23*, *rps12*, *rps19*, *rps7*, *ndhB*, and *ycf1*), seven tRNAs (*trnA-UGC*, *trnE-UUC*, *trnL-CAA*, *trnM-CAU*, *trnN-GUU*, *trnR-ACG*, and *trnV-GAC*), and four rRNAs (*rrn4.5S*, *rrn5S*, *rrn16S*, and *rrn23S*). *A. indica* var. *siamensis* do not have the *rrn4.5* gene in the IR regions, thus it has two fewer rRNA genes than *A. indica* and *A. excelsa*.

The phylogenetic results showed that three species of the genus *Azadirachta* grouped together, next to the closely related genus *Melia*. The two accessions of *A. indica* (GenBank OK037102.1 and NC023792.1) formed a clade*. A. indica* var. *siamensis* and *A. exselsa* formed a sister clade that *A. indica* was separated from these two species.

## Discussion

*A. indica* was once named *Melia azadirachta* L., and they're used to be frequent confusion between *A. indica* and the chinaberry tree (*M. azedarach* L.) in the past (Schmutterer 1995). Since the genus *Azadirachta* was established in 1830, the name has been changed to *A. indica* A. Juss (Peng et al. 2012). This work further confirms that *A. indica* taxonomically belongs to the genus *Azadirachta* at the chloroplast genome level. The complete cp genome of *A. indica* we obtained (GenBank OK037102.1) is not consistent in size with the previously measured 160,737 bp (GenBank NC023792.1). For plants, the intraspecific variation that existed in cpDNA can lead to the diversification of chloroplast gene coding traits, which is conducive to adapting to different environments (climate, soil, and biotic interactions) and facilitating evolution (Godbout et al. 2020).

Schmutterer (1995) supported the name *A. siamensis* instead of *A. indica* var. *siamensis,* because ecophysiological characteristics, phenotypic characteristics, wood anatomical characteristics and secondary metabolic compounds between *A. indica* var. *siamensis* and *A. indica* are different. According to our chloroplast phylogenetic analysis, it is also suggested that *A. siamensis* could be a species, not a variety. It was reported that individuals with intermediate morphological features of the leaflets between *A. indica* and *A. siamensis* were found in Thailand in places where both two species grow together (Schmutterer 1995). These individuals could be natural hybrids of *A. indica* and *A. siamensis*, which are not clearly defined yet. Based on the characteristic of maternal inheritance of chloroplast genomes in most angiosperms (Corriveau and Coleman 1988), it is helpful to find out the maternal source of possible hybrids.

## Data Availability

The genome sequence data that support the findings of this study are openly available in GenBank of NCBI (https://www.ncbi.nlm.nih.gov/) under the accession no. OK037100.1, OK037101.1 and OK037102.1. The associated BioProject number is PRJNA756900 and PRJNA823104, including SRA numbers (SRR16605973, SRR16605974, and SRR16605975, respectively) and Bio-Sample numbers (SAMN22566529, SAMN22566528, and SAMN20929985, respectively).
